# Application of Chitosan/Alginate Nanocomposite Incorporated with Phycosynthesized Iron Nanoparticles for Efficient Remediation of Chromium

**DOI:** 10.3390/polym13152481

**Published:** 2021-07-28

**Authors:** Fahad M. Almutairi, Haddad A. El Rabey, Adel I. Alalawy, Alzahraa A. M. Salama, Ahmed A. Tayel, Ghena M. Mohammed, Meshari M. Aljohani, Ali A. Keshk, Nasser H. Abbas, Mohamed M. Zayed

**Affiliations:** 1 Biochemistry Department, Faculty of Science, University of Tabuk, Tabuk 47512, Saudi Arabia; fahadalmutairi316@gmail.com (F.M.A.); elrabey@hotmail.com (H.A.E.R.); aalalawy@ut.edu.sa (A.I.A.); 2 Genetic Engineering and Biotechnology Research Institute, University of Sadat City, Sadat 32897, Egypt; Nasser.abbas@gebri.usc.edu.eg; 3 Department of Fish Processing and Biotechnology, Faculty of Aquatic and Fisheries Sciences, Kafrelsheikh University, Kafrelsheikh 33516, Egypt; sohairkhojah@gmail.com; 4 Department of Nutrition and Food Science, Faculty of Home Economics, University of Tabuk, Tabuk 71491, Saudi Arabia; gmohammed@ut.edu.sa; 5 Department of Chemistry, Faculty of Science, University of Tabuk, Tabuk 71491, Saudi Arabia; mualjohani@ut.edu.sa (M.M.A.); akeshk@ut.edu.sa (A.A.K.); 6 Department of Aquaculture, Faculty of Aquatic and Fisheries Sciences, Kafrelsheikh University, Kafrelsheikh 33516, Egypt; m.mamdoh7712@yahoo.com

**Keywords:** biopolymers, adsorption conditions, green synthesis, nano-conjugation, water remediation

## Abstract

Biopolymers and nanomaterials are ideal candidates for environmental remediation and heavy metal removal. As hexavalent chromium (Cr^6+^) is a hazardous toxic pollutant of water, this study innovatively aimed to synthesize nanopolymer composites and load them with phycosynthesized Fe nanoparticles for the full Cr^6+^ removal from aqueous solutions. The extraction of chitosan (Cht) from prawn shells and alginate (Alg) from brown seaweed (*Sargassum linifolium*) was achieved with standard characteristics. The tow biopolymers were combined and cross-linked (via microemulsion protocol) to generate nanoparticles from their composites (Cht/Alg NPs), which had a mean diameter of 311.2 nm and were negatively charged (−23.2 mV). The phycosynthesis of iron nanoparticles (Fe-NPs) was additionally attained using *S. linifolium* extract (SE), and the Fe-NPs had semispherical shapes with a 21.4 nm mean diameter. The conjugation of Cht/Alg NPs with SE-phycosynthesized Fe-NPs resulted in homogenous distribution and stabilization of metal NPs within the polymer nanocomposites. Both nanocomposites exhibited high efficiency as adsorbents for Cr^6+^ at diverse conditions (e.g., pH, adsorbent dose, contact time and initial ion concentration) using batch adsorption evaluation; the most effectual conditions for adsorption were a pH value of 5.0, adsorbent dose of 4 g/L, contact time of 210 min and initial Cr^6+^ concentration of 75 ppm. These factors could result in full removal of Cr^6+^ from batch experiments. The composited nanopolymers (Cht/Alg NPs) incorporated with SE-phycosynthesized Fe-NPs are strongly recommended for complete removal of Cr^6+^ from aqueous environments.

## 1. Introduction

Biopolymers are biologically synthesized macromolecules of living organisms, e.g., plants, microbes, algae and animals [1]. Many valuable biopolymers are attained from marine sources, e.g., chitosan, alginate, fucoidan, carrageenan, etc., to become extensively involved in numerous products and applications, serving environmental, biomedical, nutritional, pharmaceutical, and industrial sectors [2]. The environmental remediation using biopolymers includes the removal and reduction of pollutants like heavy metals (HM), toxic organic dyes and solvents, pesticides, and the separation of oil or other toxic pollutants from water [2,3,4,5]. 

Chitosan (Cht), “the derived cationic polysaccharide from chitin N-deacetylation”, is a linear composed biopolymer from N-acetyl-D-glucosamin and β-linked D-glucosamine; the Cht amine groups provide special features to the polymer (e.g., high reactivity, elevated charge density, ability of binding ions/compounds and biocompatibility) with biological systems [6]. The Cht biopolymers extraction could be achieved mostly from crustacean exoskeletons and was also attained from fungi mycelium and insect skeletons [7,8,9,10]. The Cht functional reactive groups (e.g., primary/secondary amines and hydroxyl groups) and their distinguishing attributes (e.g., biocompatibility, non-toxicity, biodegradability, elevated adsorption capacity, mucoadhesion, antioxidant, antimicrobial, and anticancer activities) enable their applications in numerous fields, including HM removal and water/soil remediation [2,10,11,12,13]. These advantageous attributes of Cht could be greatly enhanced by its transformation into nanoparticles (NPs) form, mainly via cross-linking with sodium tripolyphosphate, and linking the formed Cht-NPs with further bioactive particles or compounds for augmenting their combined antimicrobial, anticancerous and environmental remediation potentialities [14,15,16,17,18]. Although Cht alone was very effectual as a coagulating and flocculating agent for HM and organic matter removal from wastewater [19], the cross-linked/modified Cht had higher capacity as an HM adsorbent. This feature was confirmed while evaluating modified Cht for hexavalent chromium (Cr^6+^) adsorption [2]. Cht polymer blending was the simplified technique that was most applied for fabricating Cht and Cht-NPs based membranes for water remediation and HM removal [14,20].

Alginate (Alg) is the extracted hydrophilic polysaccharide from brown macroalgae. This linear biopolymer composition contains uronic acids, α-l-guluronic acid, and 1,4-linked-β-d-mannuronic acid [21]. Alg properties include non-toxicity, biodegradability, mucoadhesion and biocompatibility, which advocate its biomedical, nutraceutical, pharmaceutical and environmental applications [22]. 

The modification of main Alg functional groups, e.g., hydroxyl and carboxyl groups, could be gathered chemically via cross-linking, etherification, or esterification [22]; these modified groups can interact with HM through complex reaction or ion exchange. Frequently, Alg with a modified sulfonic group is prepared in hydrogel forms and composites (as active adsorption material) for HM removal and wastewater treatment [23]. 

Both Cht and Alg blends/composites, whether in bulk or in NPs form, were efficient as powerful HM adsorbents with As(V), Au(III), Cd (II), Cr(VI), Cu(II), Hg(II), Mo(V), Ni(II), Pb(II), Pd(II), Pt(IV), Se(V), U(VI) and V(V); their combined high adsorption capacities were influenced by their forms (powder, beads, sheets), chemical modifications (grafting reactions, cross-linking and encapsulation) and experimental conditions (particle size, pH, solution composition and conditioning) [22,23,24,25,26]. 

Aquatic environment contamination and pollution with HM is a serious problem globally; and overcoming this problem is very challenging [24]. Cr metal is broadly incorporated in diverse industrial practices, e.g., leather tanning, metallurgy, electroplating, etc. The Cr^6+^ forms are seriously carcinogenic, toxic (even at little doses), and possess high mobility in water [27]. 

The synthesis of nanomaterials (including nanometals and nanopolymers) and their employments in most disciplines were achieved with great success, particularly in environmental protection fields [14,27,28]. The NPs have distinguishing unique characteristics like superior size, conduction, distribution, and shape. Iron nanoparticles (Fe-NPs) have most of the favorable attributes of accustomed NPs, together with their paramagnetic, biodegradable, and biocompatible qualities, as well as non-toxicity for humans [29,30].

For nanometals synthesis, contrary to the expensive, time-consuming, and complicated features of physical methods, and hazardous residues and toxicity from chemical methods, the biosynthesis (green synthesis) methods are much safer, easier, effectual, and inexpensive methods for NPs fabrication [18]. The Fe-NPs biosynthesis was successful using biological derivatives. Promisingly, many prosperous investigations documented the algal biosynthesis (phycosynthesis) of metal NPs (including Fe-NPs), using both macro and micro algae extracts [31,32,33,34,35], which could add extra bioactivities to these phycosynthesized NPs, e.g., enhanced antiviral, antibacterial, antifungal and antitumor potentialities [36,37].

The Fe-NPs phycosynthesis was particularly advised as an environmentally-friendly, simple, low-cost and pollutant-free approach [38,39], which enabled their effectual employment for solving environmental cleanup challenges, especially with the employment of algae extracts for their phycosynthesis [32,38,40]. 

The biosynthesized Fe-NPs were enhanced via their loading, capping, and stabilizing by some biopolymers, e.g., lignin, carboxymethyl cellulose, chitosan, and alginate [28,41]; the combination of Fe-NPs with these biopolymers augmented their capabilities for removing Cr^6+^ and other HM from water [42]. However, no obtainable investigations were conducted for the phycosynthesis of Fe-NPs and their incorporation into Cht/Alg nanocomposites for effectual removal of Cr^6+^ from aqueous solutions. 

Accordingly, the experiments of the current study aimed to perform Cht and Alg extraction from marine sources, fabrication of Cht/Alg NPs composite, phycosynthesis of Fe-NPs using *Sargassum linifolium* algal extract, and evaluation of the innovative composited Cht/Alg NPs and Cht/Alg/Fe-NPs as potential active adsorbents to remove or adsorb hexavalent chromium from aqueous solutions.

## 2. Materials and Methods

### 2.1. Chemicals and Reagents 

All of the reagents and chemicals used in experimentation were certified analytical grade and were attained from Sigma-Aldrich Co. (St. Louis, MO, USA), unless other sources are reported. The chemicals and reagents included hydrochloric acid (HCl, 37%), sodium hydroxide (NaOH), acetic acid (CH₃COOH), sodium-tripolyphosphate (TPP), sodium chloride (NaCl), ethanol (C_2_H_5_OH), ferric chloride hexahydrate (FeCl_3_•6H_2_O), sulfuric acid (H_2_SO_4_, ≥97.0%), sodium carbonate (Na₂CO₃), potassium dichromate (K_2_Cr_2_O_7_, ≥99.5%), 1,5-diphenylcarbazide, and calcium chloride (CaCl_2_).

### 2.2. Biological Samples Collection

The biological samples used, e.g., prawn and macroalgae (*Sargassum linifolium*), were amassed from the Saudi western coast at the Red Sea, within E 35°65′–E 37°12′ and N 26°03′–N 31°15′. All samples were identified morphologically by specialized marine biologists at the University of Tabuk in Saudi Arabia.

### 2.3. Extraction of Marine Biopolymers

#### 2.3.1. Chitosan (Cht)

Freshly caught *Penaeus semisulcatus* (tiger-green prawn) were manually beheaded and peeled; the prawn shells were extensively rinsed with deionized water (DW), drained, and air-dried at 45 °C for 42 h. After the grinding of the dried materials, the powder was used for Cht extraction, which involved deproteinization (in 25-fold (*w/v*) 2.0 N NaOH, 6 h, 25 °C), demineralization [in 25-fold (*w/v*) 2.0 N HCl, 4 h, 25 °C], and deacetylation [in 30-fold (*w/v*) 55% NaOH solution, 115 min, 121 °C]. Each step was followed with recurrent DW washing until neutral pH was achieved. Resultant Cht was air-dried at 45 °C and characterized.

#### 2.3.2. Alginate (Alg)

The extraction of sodium alginate (Alg) was performed from *Sargassum linifolium,* using slightly modified protocols [21,43]. Briefly, collected seaweed biomass (100 g) was recurrently cleansed with DW, air-dried and soaked in 1 L of 2% formaldehyde solution for 14 h to eradicate pigments, then washed with DW and immersed in 0.2 M H_2_SO_4_ solution (at 1:10 *w/v*) before keeping for 2 h. Next, samples were further rinsed by DW and extracted with 5% Na₂CO₃ solution for 6 h under agitation. Acid and alkali treatments were each followed by centrifugation and DW washing. After obtaining Na-Alg gel, it was frozen at −18 °C, and then subjected to lyophilization. 

### 2.4. Preparation of Biopolymers Nanoparticles 

For preparing chitosan-alginate nanoparticles (Cht/Alg NPs), the microemulsion protocol was implemented [24]. Briefly, both Cht and Alg were dissolved in 2% acetic acid solution and hot DW (at 1% *w/v*), respectively, for preparing the aqueous phases. For the oil phase, paraffin oil was employed. These 3 solutions were homogenized (equal volumes, 10 mL each) for 30 min (Seward Stomacher, 400, Norfolk, UK) for forming stable emulsions, then cross-linkers were added (10 mL of Na-tripolyphosphate (1 M) and 10 mL of CaCl_2_ solutions (1 M)) with constant stirring for 180 min at 25 ± 2 °C. The pellet of NPs formed was attained by centrifugation (SIGMA, 2–16 KL, Osterode, Germany) at 12,000× *g* for 30 min, washed once with DW and twice with acetone, then recentrifuged and the acquired NPs pellet was lyophilized and analyzed.

### 2.5. Iron Nanoparticle Phycosynthesis

The *S. linifolium* algal extract (SE) was prepared using ethanol (70%) as a solvent. First, 50 g of algae powder was immersed in 500 mL of solvent and stirred for 8 h at 320× *g* at 25 ± 2 °C. The SE was attained by paper filtration to omit algal residues, then the solvent was discarded via vacuum evaporation and the dried SE was re-constituted in 10 folds of DW. For Fe-NPs biosynthesis, 0.1 M solution of FeCl_3_·6H_2_O was dropped slowly (0.5 mL/min) into SE at a 1:2 ratio (*v/v*) of FeCl_3_ solution:SE, stirred at 425 × *g,* and the stirring was extended for extra 90 min after dropping. The change of the solution color to black indicated the NPs phycosynthesis. The reaction solution was quickly centrifuged at 14,000× *g* for 33 min and the NPs pellets were resuspended in DW, frozen at −20 °C and lyophilized [44].

### 2.6. Fabrication of Nano-Adsorbents

Aqueous solutions (10 mg/mL) from Cht/Alg NPs and SE-synthesized Fe-NPs were prepared using vigorous homogenization of nanomaterials in DW until achieving clear solutions, which were additionally filtered to eliminate any undisclosed particles. The Cht/Alg NPs solution were stirred 450× *g* at 25 ± 2 °C and the SE-synthesized Fe-NPs solution (equal volume) was dropped slowly into biopolymers solution under stirring for 120 min. Subsequently, the formed Cht/Alg/Fe-NPs composited particles were separated with centrifugation, washed with DW, frozen and lyophilized. 

### 2.7. Characterization of Products and Nanomaterials

#### 2.7.1. FTIR Analysis

The FTIR (Fourier Transform Infrared Spectrophotometer, Perkin Elmer 200, Waltham, MA, USA) was applied for analyzing the biochemical bonding in produced/fabricated materials, including the SE, SE-synthesized Fe-NPs, Cht, Alg, and Cht/Alg NPs. The measurements of samples, after mixing with KBr, were recorded at 25 °C in a wavelength range of 450–4000 cm^−1^ with 4 cm^−1^ resolution.

#### 2.7.2. Structural Analysis of Nanometals

The TEM (transmission electron microscopy, Leo 0430, Leica, Cambridge, UK) imaging was employed for assessing the structural features size, morphology, shape, and distribution of SE-phycosynthesized Fe-NPs.

#### 2.7.3. SEM (Scanning Electron Microscopy) Imaging

The surface structural morphology of the composited Cht/Alg/Fe-NPs was witnessed via SEM (JEOL JSM- IT100, Tokyo, Japan) to validate the composite compatibility and physiognomy. 

#### 2.7.4. Particles’ Size and Charge 

The detection of particle size (Ps) distribution of synthesized nanomaterials/nanocomposites (e.g., Fe-NPs, Cht/Alg NPs and Cht/Alg/Fe-NPs) and their surface charges (Zeta potential) were conducted via DLS (Dynamic Light Scattering, Malvern Zetasizer, Malvern Instruments, Malvern, UK).

### 2.8. Batch Adsorption of Hexavalent Chromium 

Batch adsorption experiments were conducted using varying conditions (e.g., pH, adsorbent dose, contact time (CT) and initial ion concentration) to evaluate the potentiality of Cht/Alg NPs and Cht/Alg/Fe-NPs to adsorb hexavalent chromium (Cr^6+^) from aqueous media [22]. The Cr^6+^ aqueous solutions (different concentrations) were prepared via dissolving K_2_Cr_2_O_7_ in buffered DW with acetate buffer. The experimented levels from each factor were: for pH. 3–8; for the adsorbent dose, 1–7 g/L; for the contact time, 30–270 min; and for the initial ion concentration, 10–150 ppm. While experimenting a factor, the rest conditions were kept at constant rates. After equilibrium attainment for each trial, the adsorbent was disjointed via vacuum filtration. The initial and final concentrations of Cr^6+^ in filtrates were quantified using FAAS (flame atomic absorption spectrometer, AA-680, Shimadzu, Kyoto, Japan). The residual Cr^6+^ concentrations were further determined accurately using 1,5-diphenylcarbazide that forms a reddish-violet complex with Cr^6+^. The absorbance of the complex was measured via UV–vis spectrophotometer (UV-2600, Shimadzu, Kyoto, Japan) at 540 nm, compared to standard calibration curves. 

## 3. Results and Discussion

### 3.1. Phycosynthesis and Characterization of Fe-NPs Using Sargassum Linifolium Extract

**Figure 1:**
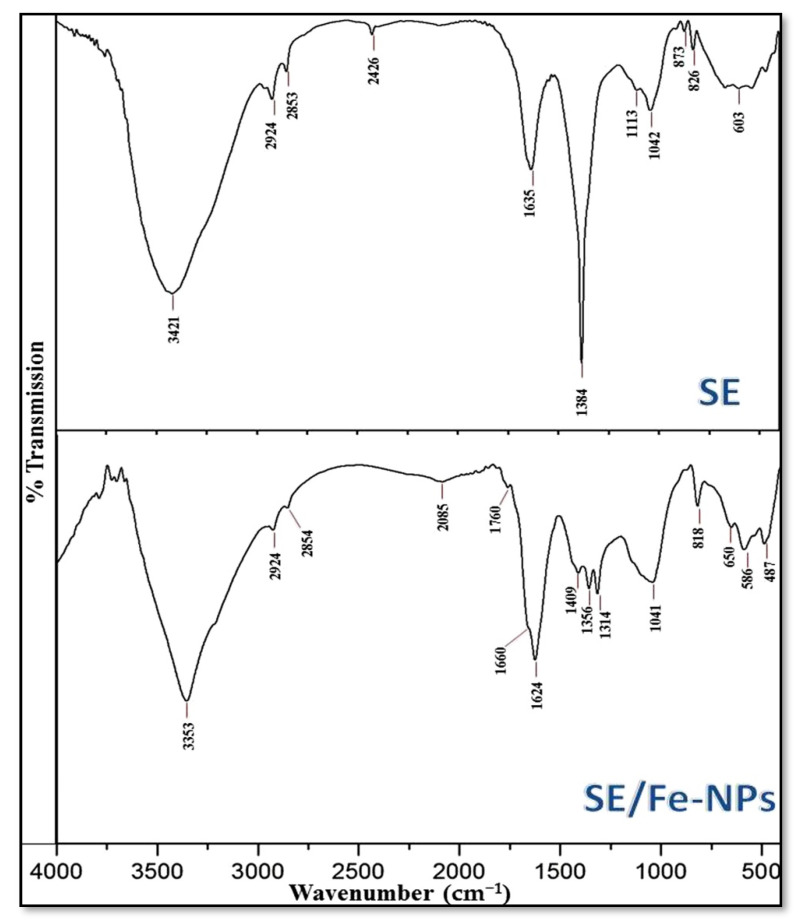
FTIR spectra of *Sargassum linifolium* extract (SE) and the combined extract with phycosynthesized Fe-NPs (SE/Fe-NPs).

The FTIR spectral analysis of SE and SE/Fe-NPs revealed the active characteristic bonds that are involved in SE biochemical activity and their potential role in Fe-NPs synthesis (Figure 1). The SE spectrum (Figure 1 SE) indicated a strong O–H absorption band at 3421 cm^−1^, related to extensive presence of this group in SE phenols and in amine groups, namely hydroxyl amide I, amide II and primary amines, on the *Sargassum* sp. walls [37]. 

The moderate peak at 2924 cm^−1^ reflected the stretched N–H bonding and/or the CH_2_/CH_3_ and vibrated stretching in SE aldehydes and aliphatic groups. The intense signal at 1635 cm^–1^ is assumingly corresponding to stretched C=O and N=O in SE esters and pectin. The absorption bands at frequencies of 1635 and 1384 cm^–1^ “indicating the combined vibrated stretching of C–O and C–C ring of phenyl”, 1042 cm^–1^ “indicating stretching S=O”, and the recorded vibrations signals at 1113 and 829 cm^−1^ “indicating the existence of SE aromatic polyphenols”, validated the main SE biochemical active bonding [45,46]. 

For the SE/Fe-NPs spectrum (Figure 1 SE-Fe-NPs), the O–H indicative peak shifted to 3353 cm^−1^ after Fe-NPs phycosynthesis. Additionally, the shifting and intense variations in SE beaks after Fe-NPs synthesis (from 1635 to 1624 cm^−1^, from 829 to 818 cm^−1^ and from 1384 to 1356 cm^−1^), and the disappearance of the indicative peak at 1113 cm^−1^, revealed the involvement of these biochemical bonds in Fe-NPs phycosynthesis and capping [32]. The “Van der Waals” interaction forces are suggested as the main causes of interference between the O and N atoms in algal extract and the phycosynthesized Fe-NPs [31].

**Figure 2:**
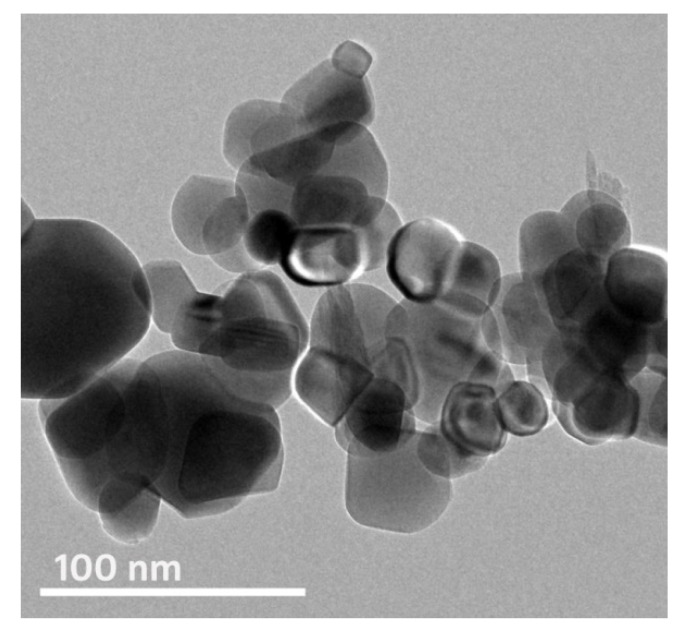
TEM imaging of phycosynthesized Fe-NPs using *Sargassum linifolium* extract.

The ultrastructure of phycosynthesized Fe-NPs was elucidated via TEM imaging (Figure 2). The image displayed the majority of NPs in spherical/semispherical shapes, with apparent agglomeration within synthesized particles. The estimated Ps of the phycosynthesized Fe-NPs was in the 13.6−72.4 nm range. The NPs agglomeration is basically attributed to the characteristics of SE, e.g., the thickening features of seaweed extract and the existence of intense O–H groups in it [30]. Moreover, the Fe-NPs agglomeration tendency was somewhat expected because of their minute sizes and potential magnetic characteristics [30,32,47].

### 3.2. Characterization of Biopolymers Nanocomposites

#### 3.2.1. FTIR Analysis

**Figure 3:**
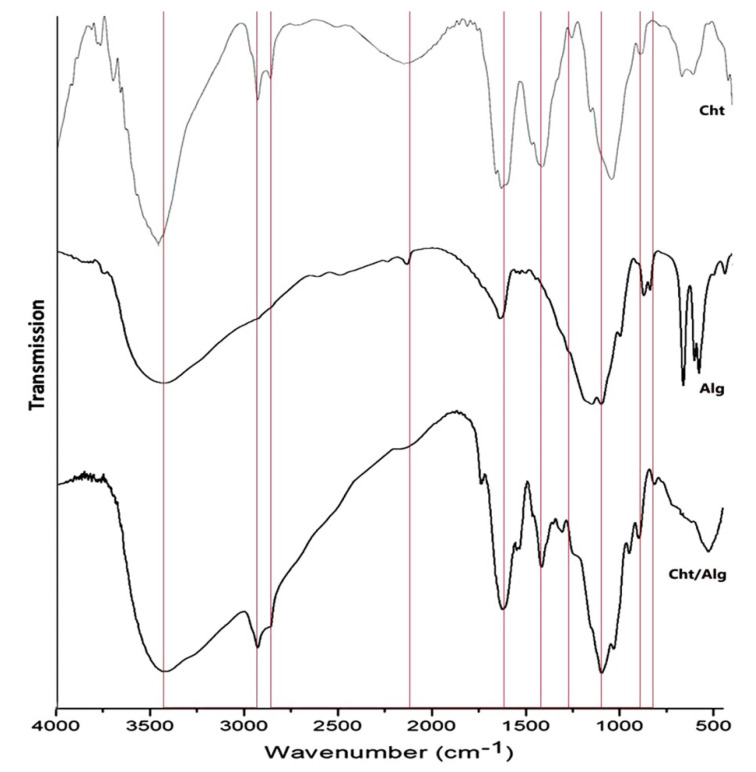
FTIR spectra of extracted chitosan (Cht), alginate (Alg) and composited chitosan/alginate nanoparticle (Cht/Alg)*.* The interacted groups/bonds from original polymers in Cht/Alg are indicated by vertical red lines.

The FTIR spectra of Cht, Alg and their composites (Cht/Alg) are offered in Figure 3. The Cht spectrum (Figure 3-Cht) appointed the existence of the key Cht characteristic groups for amide III, amide I and N–H, and at absorption wavelength of 1425, 1671, and 3418 cm^−1^, respectively. The band at 1031 cm^−1^ corresponds to C–O and C–C stretching [48].

The distinguishing peaks of Alg (Figure 3-Alg) represented a stretching O–H bond at 3396 cm^−1^, asymmetric COO– stretching at 1619 and 1430 cm^−1^, and stretched C–O–C at 1016 cm^−1^ [49]. The FTIR spectra of extracted Cht and Alg are strongly comparable to the standardized commercial sample of these biopolymers [48,50].

In Cht/Alg (Figure 3-Cht/Alg), the composite spectrum had the most distinctive groups from each of the individual polymers, which validated the polymers crosslinking and biochemical interactions. Additionally, the shifting of C–O–C stretching signal (in Alg spectrum) from 1016 to 1028 cm^−1^ indicated the cross-linkage of Alg with Ca^2+^ and the ionic bonding between Alg carboxyl groups and calcium ions [41,50]. As observed after Cht/Alg cross-link interactions, the stretched –CH vibration peaks of Cht and Alg overlapped; more hydrogen bonds were formed between Cht amino groups “transformed to NH^3+^ and produced polyelectrolyte compound under strong electrostatic interaction” and Alg carboxyl anions [51].

#### 3.2.2. SEM Imaging

**Figure 4:**
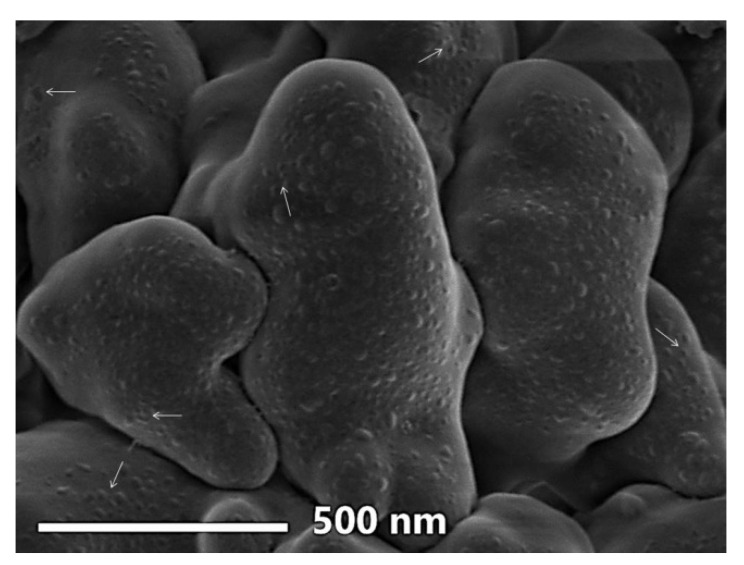
SEM image of constructed chitosan/alginate nanocomposite with phycosynthesized Fe-NPs*. * Arrows indicate examples of incorporated Fe-NPs to Cht/Alg nanocomposites.

The ultrastructure imaging of constructed Cht/Alg nanocomposite with SE-phycosynthesized Fe-NPs is elucidated in Figure 4. The nanocomposite had ununiformed shapes with estimated Ps of 186.1−542.7 nm range. The incorporated Fe-NPs to Cht/Alg nanocomposite were notably detectable up to the surfaces of nanoparticles, which indicates their potentially strong capability for interacting with the surrounding environment and its components. Additionally, the Fe-NPs appeared in non-agglomerated forms, which confirmed the potency of Cht/Alg nanocomposite for capping and stabilizing the SE-phycosynthesized Fe-NPs [40,52,53].

#### 3.2.3. Nanomaterials’ Size and Charge 

The analysis of Ps and charge of fabricated NPs indicated the minute Ps range of Fe-NPs (12.5–83.1 nm), with a mean Ps diameter of 21.4 nm (Table 1). The Ps ranged from 162.6–514.6 for Cht/Alg NPs and from 173.9–568.5 for Cht/Alg/Fe nanocomposite, with mean diameters of 311.2 and 342.6 nm, respectively. The integration of Fe-NPs into the polymers’ nanocomposite led to a slight increasing in Ps and remarkable increment in nanocomposite negative charging from −23.2 to −34.6 mV (Table 1).

### 3.3. Batch Adsorption of Cr(VI)

**Figure 5:**
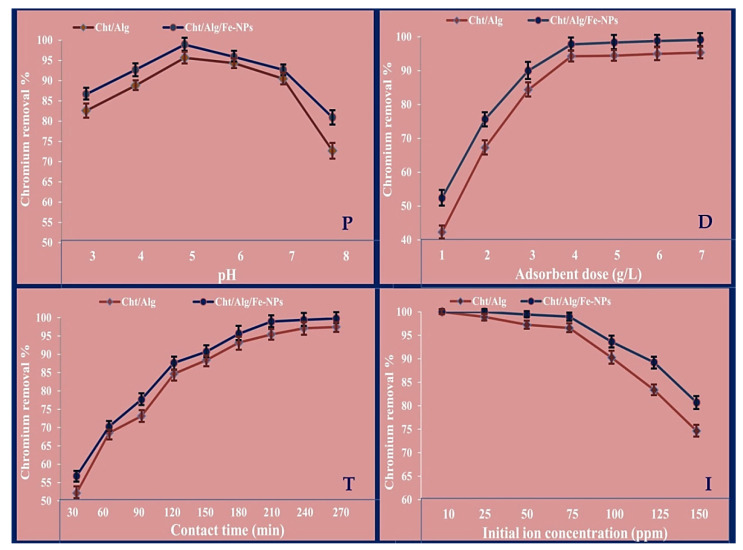
Effect of batch adsorption conditions, including pH (P), adsorbent dose (D), contact time (T) and initial ion concentration (I), on chromium removal (%) using chitosan/alginate nanoparticles and chitosan/alginate/iron nanocomposite*. * The vertical bars in curves indicate the standard deviation of results.

The batch adsorption of Cr(VI) was successfully and effectually achieved, using both Cht/Alg NPs and composited Cht/Alg/Fe-NPs, with 100% removal percentages in some experiments (Figure 5). While examining a factor, the rest conditions were kept at constant rates (e.g., pH = 5, adsorbant dose = 5 g/L, contact time = 200 min and initial ion concentration = 100 ppm). Generally, the composited Cht/Alg/Fe-NPs had higher efficiency of Cr(VI) adsorption than Cht/Alg NPs with each parameter. Due to higher contents from −OH (hydroxyl groups) and–NH_2_ (primary amine), which act as powerful sorption sites, Cht-based compositions were advocated as excellent materials for the adsorbing and removal of many heavy metals, including Cr(VI), from solution [54]. The transformation of biopolymers, e.g., Cht and Alg, into nano-forms greatly increased their potentialities and removal capacity of heavy metals due to their enhanced physiochemical properties, providing larger contact area, more functioning groups, and extra sorption sites [53]. These parameters increased with the loading of nanopolymers with metals (e.g., iron nanoparticles), which may explain the higher capacity of Cht/Alg/Fe-NPs composite for absorbing/removing Cr(VI) [44,55].

The consequence of pH variation on Cr^6+^ adsorption by Cht/Alg NPs and Cht/Alg/Fe-NPs is obtainable from Figure 5P, at a pH range of 3–8. The highest Cr^6+^ removal (%) were 95.66% and 98.90% using Cht/Alg NPs and Cht/Alg/Fe-NPs, respectively, at pH 5. The metal solution pH can affect the surface charging of adsorbents speciation; the active biochemical sites of adsorbents are either deprotonated or protonated with pH alterations, and consequently the ions’ adsorption is affected [56]. At lower examined pH values, less Cr(VI) ion uptake was detected, assumingly because of the competitive H^+^ and Cr^6+^ adsorption onto the polymers’ nanocomposites surface. The protonation of polymer molecules, at lower pH values, can reduce the amount of available sites for metal ion binding. Furthermore, amino groups’ protonation generates electrostatic repulsions on metallic cations, henceforth decreasing accessible sites for ion adsorption [57]. The Cr(VI) adsorption increased with pH increment, up to pH = 5, because more active adsorption sites on polymer nanocomposites were deprotonated and had more net attraction forces that enabled higher Cr adsorption from solution. The optimum pH for Cr^6+^ adsorption was 5, then further pH increments led to lower ion adsorption, mostly because the precipitation of Cr(OH)_3_ complexes, which hinders the adsorption development. The adsorption reduction in higher pH could be a result of development of further solubilized hydroxyl complexes [25]. Although Fe-NPs surface modifications could prevent their oxidation, especially when barriers between target pollutants and Fe-NPs are created which may influence Fe-NPs ability and reactivity for pollutant’ removal, the employment of modified coating materials with high reactivity for HM removal can increase the removal potentialities of their composites [53]. The oxidation/reduction potential of Cr^6+^ ions was illustrated to increase with pH decline; Cr^6+^ ions became more susceptible to reduction in acidic solutions [57]. As Cht and Alg were evidenced as excellent HM adsorption material, they exhibited good affinities for Cr^6+^ removal, either as plain composite, or after as active coating for adsorbing NPs [25,54]. Thus, the composited mixture from Cht/Alg/Fe-NPs, and the examined pH values for Cr^6+^ adsorption trials, could control the oxidation potentiality of Fe-NPs

The adsorbent dose consequence on Cr^6+^ adsorption was investigated at 1−7 g/L ratios of Cht/Alg NPs and Cht/Alg/Fe-NPs composite in metal solution (Figure 5D). This evidenced that Cr^6+^ adsorption was at first speedily increased with adsorbent dose increment up to 4 g/L dosage, then remained mostly constant. The highest Cr^6+^ removal (%) were 94.26% and 97.78% using Cht/Alg NPs and Cht/Alg/Fe-NPs, respectively, at 4 g/L dose. The initial prompt Cr uptake indicated high affinities between the adsorbate and the adsorbent nanocomposites, which are accredited to the adsorbents’ biochemical characteristics. The extra addition of the adsorbents beyond 4 g/L did not trigger significant adsorption increment, which is assumed to be attributed to adsorbent particles overcrowding [24,57]. At the lower concentrations, adsorbent nanocomposites had sufficient surface area, adsorption sites, and amount of Fe-NPs for Cr^6+^ uptake [58], whereas at higher adsorbent doses, the adsorption sites of nanopolymers composites are overlapping and their particles are overcrowding, which are suggested to decrease ions adsorption [25,53].

The contact time (CT) is an influential factor in batch adsorption practice; the CT influence on Cr^6+^ adsorption, by Cht/Alg NPs and Cht/Alg/Fe-NPs composite, is presented in Figure 5T. The Cr^6+^ adsorption experiment was prolonged from 30−270 min, with 30 min intervals. Results indicated that Cr^6+^ adsorption significantly increased with CT prolongation up to 210 min, then the increment of removal percentage became insignificant. The highest Cr^6+^ removal (%) were 95.42% and 98.93%, using Cht/Alg NPs and Cht/Alg/Fe-NPs, respectively, after a CT of 210 min. Initially, the higher Cr adsorption rate was possibly attributed to large available surface area from polymer composite to adsorb the ions. Subsequently, due to exhaustions of nanopolymers adsorption sites, no improvements could be achieved in Cr^6+^ adsorption with CT prolongation beyond 210 min [59]. 

The effect of initial Cr^6+^ concentration on the adsorption potentialities of Cht/Alg NPs and Cht/Alg/Fe-NPs composite is presented in Figure 5I. Both Cht/Alg NPs and Cht/Alg/Fe-NPs could absorb/remove 100% from Cr^6+^ at 10 ppm ion concentration. At higher concentrations (25, 50, 75 and 100 ppm), the removal percentages were 98.9%, 97.23%, 96.55% and 90.24% for Cht/Alg NPs, and 100%, 99.42%, 98.96% and 93.61% for Cht/Alg/Fe-NPs composite, respectively. This could be simply explained by the ability of the nanopolymers’ composites to possess sufficient binding/adsorption sites to uptake most orall Cr^6+^ ions in beginning concentrations. Then, these sites possess partial saturation with metal ions at higher concentrations, which can somewhat lessen their removal capabilities [60,61].

## 4. Conclusions 

The extraction of Cht from prawn shells and Alg from brown seaweed (*Sargassum linifolium*) was achieved with standard characteristics, the tow biopolymers was combined and cross-linked to generate Cht/Alg NPs, which had a mean diameter of 311.2 nm. The phycosynthesis of Fe-NPs was successfully attained using *S. linifolium* extract (SE) and the Fe-NPs had semispherical shapes with a 21.4 nm mean diameter. The conjugation of Cht/Alg NPs with SE-phycosynthesized Fe-NPs resulted in homogenous distribution and stabilization of metal NPs within the polymer nanocomposites. Both nanocomposites exhibited high efficiency as adsorbents for Cr^6+^ at diverse conditions; the most effectual conditions for adsorption were a pH value of 5.0, adsorbent dose of 4 g/L, contact time of 210 min and initial Cr^6+^ concentration of 75 ppm. These factors could result in the full removal of Cr^6+^ from batch experiments. The composited nanopolymers (Cht/Alg NPs), which were innovatively incorporated with SE-phycosynthesized Fe-NPs, are strongly recommended for complete removal of Cr^6+^ from aqueous environments. Subsequently, these effectual nanocomposited sorbents are candidates for further HM sorption trials toward extra pollutants and process optimizations.

## Figures and Tables

**Table 1:** Particle size and charging analysis of the produced nanomaterials including phycosynthesized Fe-NPs, chitosan/alginate NPs and chitosan/alginate/Fe nanocomposite.

Nanoparticles	Particles Range (nm)	Mean Diameter (nm)	Median Diameter (nm)	Zeta Potential (mV)
Fe-NPs	12.5–83.1	21.4	22.1	−25.6
Cht/Alg NPs	162.6–514.6	311.2	332.6	−23.2
Cht/Alg/Fe NPs	173.9–568.5	342.6	382.5	−34.6

## Data Availability

The data presented in this study are available on request from the corresponding author.
