# Correction: Almutairi et al. Application of Chitosan/Alginate Nanocomposite Incorporated with Phycosynthesized Iron Nanoparticles for Efficient Remediation of Chromium. *Polymers* 2021, *13*, 2481

**DOI:** 10.3390/polym14071385

**Published:** 2022-03-29

**Authors:** Fahad M. Almutairi, Haddad A. El Rabey, Adel I. Alalawy, Alzahraa A. M. Salama, Ahmed A. Tayel, Ghena M. Mohammed, Meshari M. Aljohani, Ali A. Keshk, Nasser H. Abbas, Mohamed M. Zayed

**Affiliations:** 1 Biochemistry Department, Faculty of Science, University of Tabuk, Tabuk 47512, Saudi Arabia; fahadalmutairi316@gmail.com (F.M.A.); elrabey@hotmail.com (H.A.E.R.); aalalawy@ut.edu.sa (A.I.A.); 2 Genetic Engineering and Biotechnology Research Institute, University of Sadat City, Sadat 32897, Egypt; Nasser.abbas@gebri.usc.edu.eg; 3 Department of Fish Processing and Biotechnology, Faculty of Aquatic and Fisheries Sciences, Kafrelsheikh University, Kafrelsheikh 33516, Egypt; sohairkhojah@gmail.com; 4 Department of Nutrition and Food Science, Faculty of Home Economics, University of Tabuk, Tabuk 71491, Saudi Arabia; gmohammed@ut.edu.sa; 5 Department of Chemistry, Faculty of Science, University of Tabuk, Tabuk 71491, Saudi Arabia; mualjohani@ut.edu.sa (M.M.A.); akeshk@ut.edu.sa (A.A.K.); 6 Department of Aquaculture, Faculty of Aquatic and Fisheries Sciences, Kafrelsheikh University, Kafrelsheikh 33516, Egypt; m.mamdoh7712@yahoo.com

The authors wish to make the following corrections to the published paper [1]. They should be as follows:

In the original publication, the funding section “**Funding**: This research was funded by Deanship of Scientific Research, University of Tabuk, KSA, under the research grant no. S-1442-0013.” should be changed to “**Funding**: The authors extend their appreciation to the Deputyship for Research & Innovation, Ministry of Education in Saudi Arabia for funding this research work through the project number (0013-1442-S).” The authors apologize for any inconvenience caused and state that the scientific conclusions are unaffected. The original publication has also been updated.
